# Dysregulated Resolution of Inflammation After Respiratory Viral Infections: Molecular Pathways Linking Neuroinflammation to Post-Viral Neuropathic Pain—A Narrative Review

**DOI:** 10.3390/ijms262311383

**Published:** 2025-11-25

**Authors:** Andrei Emilian Popa, Elena Popa, Tatiana Dramba, Elena Adorata Coman, Mihaela Poroch, Monica Ungureanu, Agnes Bacusca, Ana Maria Slanina, Gema Bacoanu, Vladimir Poroch

**Affiliations:** 1 Faculty of Medicine, “Grigore T. Popa” University of Medicine and Pharmacy, 700115 Iasi, Romania; andreiemilianpopa@gmail.com; 2 2nd Internal Medicine Department, “Grigore T. Popa” University of Medicine and Pharmacy, 700115 Iasi, Romania; gema.bacaoanu@umfiasi.ro; 3 Preventive Medicine and Interdisciplinarity Department, “Grigore T. Popa” University of Medicine and Pharmacy, 700115 Iasi, Romania; elena.popa@umfiasi.ro (E.P.); elena.coman@umfiasi.ro (E.A.C.); boanca.mihaela@umfiasi.ro (M.P.); agnes.bacusca@umfiasi.ro (A.B.); ana_slanina@umfiasi.ro (A.M.S.); vladimir.poroch@umfiasi.ro (V.P.)

**Keywords:** viral infection, neuroinflammation, resolution of inflammation, neuropathic pain

## Abstract

Post-viral neuroinflammatory syndromes, particularly those occurring after SARS-CoV-2 infection, have received increasing attention due to their complex and persistent neurological manifestations. The aim of this narrative review is to integrate current evidence on the molecular and cellular mechanisms underlying chronic neuroinflammation following viral infections, with a focus on dysregulated innate immune responses, macrophage–microglia interactions, oxidative–mitochondrial stress, and impaired inflammation resolution pathways. Our synthesis shows that prolonged activation of macrophages and glial cells promotes the continuous release of pro-inflammatory mediators, while defective phagocytosis and inadequate clearance of cellular debris maintain an inflammatory microenvironment. Mitochondrial dysfunction further amplifies immune activation by stimulating metabolic stress and reactive oxygen species production. In parallel, deficiencies in mediators specialized in inflammation resolution impede the transition from inflammation to resolution, allowing neuroimmune imbalance and nociceptive sensitization to persist long after virus clearance. Key conclusions indicate that these interconnected mechanisms collectively contribute to the long-term neurological symptoms observed in post-viral states, including cognitive impairment, neuropathic pain, and fatigue. Emerging therapeutic strategies targeting cytokine signaling, microglial reactivity, mitochondrial function, and resolution pathways are promising, but remain insufficiently validated in clinical practice. Overall, evidence suggests that post-viral neuroinflammation results from the convergence of sustained immune activation and failure of endogenous resolution mechanisms, highlighting the need for further mechanistic studies and targeted interventions.

## 1. Introduction

Inflammation is a fundamental innate immune response, essential for eliminating pathogens and harmful stimuli, as well as for restoring tissue homeostasis. Traditionally, the resolution of inflammation was considered a passive process, resulting from the dissipation of pro-inflammatory mediators [1,2,3]. However, recent research has demonstrated that resolution is an active, tightly regulated molecular and cellular process. It involves macrophage reprogramming through efferocytosis, post-transcriptional regulatory mechanisms, and the synthesis of specialized pro-resolving lipid mediators (SPMs), such as lipoxins, resolvins, protectins, and maresins [2,4]. These molecules promote the clearance of apoptotic cells and tissue debris, inhibit excessive neutrophil recruitment and facilitate the transition toward reparative macrophage phenotypes, thereby preventing the progression from acute to chronic inflammation [2].

In some individuals, dysregulation or delay of the resolution phase results in a state of persistent low-grade inflammation [3]. This maladaptive response extends beyond the respiratory tract, disrupting peripheral and central nervous system (CNS) homeostasis and fostering chronic neuroinflammation [5]. Respiratory viruses are of particular relevance in this context because they induce robust epithelial and innate immune activation in airways, leading to the release of cytokines, chemokines, and epithelial alarmins that disseminate systemically, disrupt blood–brain barrier integrity, and prime microglia [5]. These mechanisms have been documented after infections with influenza viruses, Epstein–Barr virus, and especially SARS-CoV-2 [5]. Given the ability of respiratory viruses to combine epithelial injury with systemic immune activation, blood–brain barrier disruption, and glial priming, they represent a uniquely powerful model for studying how unresolved inflammation progresses toward neuroinflammation and neuropathic pain (NP) [5,6].

Clinically, these pathways are increasingly recognized in the neurologic post-acute sequelae of SARS-CoV-2 infection (Neuro-PASC), a neurological phenotype within the broader spectrum of long COVID and in other post-viral syndromes characterized by fatigue, cognitive dysfunction, anosmia, and NP [6].

These clinical patterns highlight the need to clarify the mechanisms sustaining persistent inflammation after respiratory viral infections. Understanding the molecular mechanisms that link impaired resolution of inflammation with post-viral NP is essential for identifying novel therapeutic targets and preventing long-term sequelae [5]. This review integrates mechanistic insights and clinical observations to provide a framework for addressing neuroinflammatory states after respiratory viral infections.

The discovery of these mechanisms has led to a paradigm shift from conventional anti-inflammatory strategies (such as corticosteroids, nonsteroidal anti-inflammatory drugs, or anti-cytokine therapies) [7] toward resolution-oriented therapies, termed resolution pharmacology [8]. These approaches not only suppress inflammation but also stimulate endogenous mechanisms that restore homeostasis, with the potential to provide superior or synergistic benefits compared to classical treatments.

Over the past decades, significant progress has been made in clarifying the phases of the acute inflammatory response and resolution: initiation of inflammation through the recognition of damage-associated molecular patterns (DAMPs) and pathogen-associated molecular patterns (PAMPs), activation of antimicrobial mechanisms, suppression of pro-inflammatory mediators, active resolution through efferocytosis, and SPM production, followed by the post-resolution phase characterized by adaptive immune cell infiltration and the establishment of immune memory [2,3,9]. The current therapeutic gap in the management of infections is the absence of agents capable of reactivating inflammatory resolution, which has increased interest in SPMs such as lipoxins and resolvins [10]. Importantly, after respiratory viral infections, failure of this resolution process establishes a pathogenic trajectory from acute inflammation to chronic low-grade inflammation, neuroinflammation, and ultimately NP [5].

In this context, increasing attention has been directed toward biomarkers capable of capturing maladaptive innate immune activation and impaired inflammatory resolution following viral infections [5,8]. Biomarkers reflecting monocyte–macrophage activation—such as the soluble form of the CD14 receptor, including its proteolytic fragment presepsin (sCD14-ST)—reflect amplified pattern-recognition signaling, inefficient clearance of DAMPs and PAMPs and sustained innate immune activity [10]. These annormalities converge with reduced SPMs biosynthesis or receptor signaling, both of which are essential for promoting efferocytosis and terminating inflammatory responses. The combined imbalance between heightened pro-inflammatory signaling and insufficient resolution fosters a pro-inflammatory milieu conducive to persistent low-grade inflammation and increases susceptibility to neuroimmune dysregulation after respiratory viral infections [5,8].

**Figure 1:**
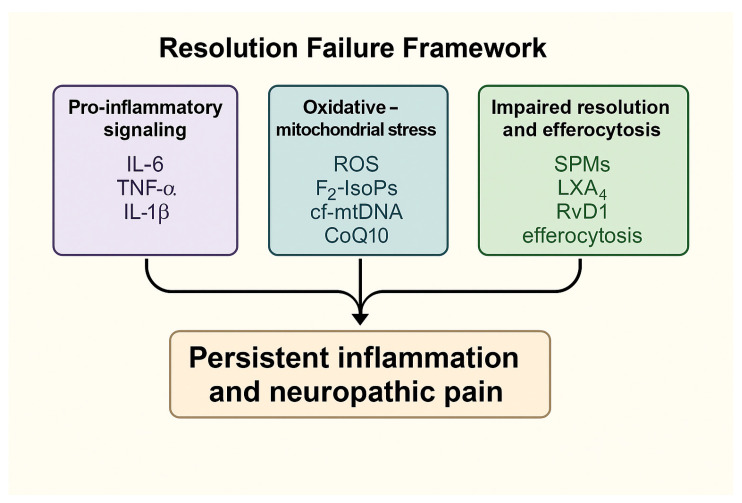
Schematic representation of the *Resolution Failure Framework*, illustrating how pro-inflammatory signaling, oxidative–mitochondrial stress and impaired pro-resolving mediator activity converge to promote persistent inflammation and neuropathic pain. Abbreviations: ROS—reactive oxygen species; F_2_-IsoPs—F_2_-isoprostanes; cf-mtDNA—cell-free mitochondrial DNA; CoQ10—coenzyme Q10; LXA_4_—lipoxin A4; RvD1—resolvin D1.

Understanding these interconnected processes defines the mechanistic continuum from acute viral-induced inflammation to chronic neuroinflammatory and neuropathic states. These interactions are summarized in the Graphical Abstract (Figure 1), which integrates the principal molecular pathways within the proposed Resolution Failure Framework.

## 2. Material and Methods

A targeted literature search was conducted in PubMed, Scopus, and Web of Science using the keywords “viral infection”, “neuroinflammation”, “resolution of inflammation”, and “neuropathic pain”. The search covered publications from 2020 to 2025, complemented by earlier foundational studies when relevant to the topic.

Studies were included if they investigated molecular, cellular, or physiological mechanisms linking viral infection—or, when mechanistically relevant, vaccination—to neuroinflammation, impaired inflammatory resolution, or neuropathic pain. Articles lacking mechanistic, translational, or clinical relevance, as well as non-peer-reviewed reports, were excluded.

A total of 152 articles met inclusion criteria: 91 experimental or clinical studies, 29 reviews, 12 meta-analyses, and 20 computational or AI-based papers. Evidence synthesis was organized around three major biological axes: pro-inflammatory signaling, oxidative–mitochondrial stress, and pro-resolving pathways.

This manuscript is designed as a narrative review and was developed in accordance with the SANRA (*Scale for the Assessment of Narrative Review Articles*) methodological framework [11], which provides structured criteria to ensure conceptual clarity, analytical coherence, and scientific rigor. Ethical approval was not required, as the review is based exclusively on the published literature.

## 3. Molecular Mechanisms of Dysregulated Inflammation Resolution

A comprehensive understanding of inflammation requires not only a description of its clinical and cellular manifestations, but also the identification of molecular control points that regulate its resolution [1]. The acute inflammatory response proceeds through sequential phases—initiation, suppression of pro-inflammatory mediators, active resolution, and post-resolution—each governed by specific mediators and signaling pathways [12]. Disruption of these tightly coordinated processes impedes the restoration of homeostasis and promotes persistent low-grade inflammation [5]. The following subsections highlight the major molecular defects underlying dysregulated resolution after respiratory viral infections.

### 3.1. Defective Efferocytosis

**Figure 2:**
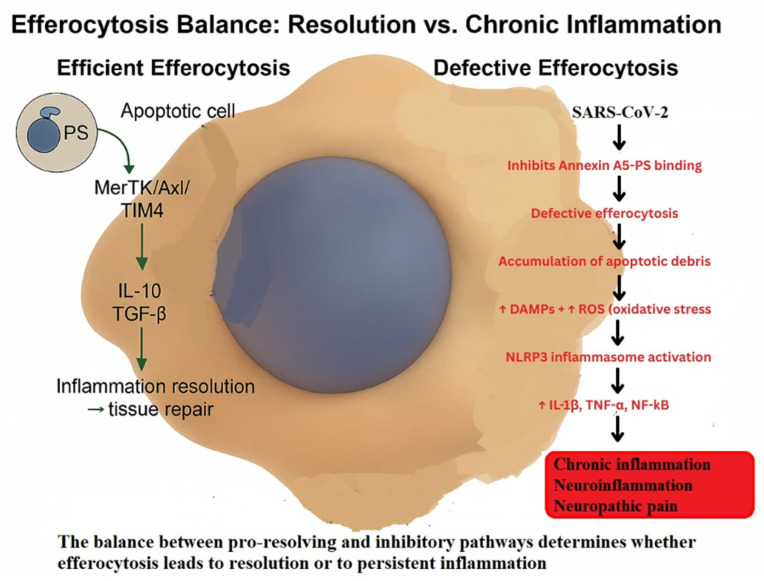
Efferocytosis and inflammation resolution—from viral infection to neuroinflammation and NP.

Efferocytosis, defined as the specialized form of phagocytosis responsible for the recognition and internalization of apoptotic cells by macrophages, represents a key critical determinant of inflammation resolution [13]. This process is mediated by a broad spectrum of receptors, Mer receptor tyrosine kinase (MerTK), Axl receptor tyrosine kinase (Axl), T-cell immunoglobulin, and mucin domain-containing protein 4 (TIM-4), which detect the externalization of phosphatidylserine (PS) on apoptotic cells and activate downstream cascades involving phosphoinositide 3-kinase–Akt (PI3K–Akt), extracellular signal-regulated kinase (ERK) and peroxisome proliferator-activated receptor gamma (PPARγ) [14,15,16,17,18]. These pathways collectively induce anti-inflammatory transcriptional programs and reparative responses. When efferocytosis fails, apoptotic cells undergo secondary necrosis, releasing DAMPs that amplify NF-κB signaling and sustain leukocyte recruitment, thereby perpetuating inflammation [19]. The key molecular mechanisms contrasting physiological and defective efferocytosis and their implications for inflammation resolution and NP are depicted in Figure 2.

Schematic representation of the transition from efficient to defective efferocytosis. Under physiological conditions, MerTK–Axl–TIM-4 signaling promotes IL-10 and TGF-β production, driving anti-inflammatory reprogramming, inflammation resolution, and tissue repair. In pathological contexts such as SARS-CoV-2 infection, inhibition of Annexin A5–phosphatidylserine binding impairs efferocytosis, leading to apoptotic debris accumulation, oxidative stress (↑ROS), DAMP release, and NLRP3 inflammasome activation. These processes sustain IL-1β, TNF-α, and NF-κB signaling, resulting in chronic inflammation, neuroinflammation, and NP [14,15,16,17,18,19].

The effectiveness of efferocytosis relies on a tightly regulated equilibrium between stimulatory and inhibitory mechanisms. Among the positive regulators, interleukin-4 (IL-4) and interleukin-13 (IL-13) promote STAT6- and 15-lipoxygenase-dependent synthesis of endogenous PPARγ ligands, including 13-hidroxioctadecadienoic acid (13-HODE) and 15-hidroxieicosatetraenoic acid (15-HETE) [13,20]. These mediators enhance the expression of phagocytic receptors such as Stabilin-1, Stabilin-2, and MerTK, while promoting secretion of bridging molecules like adiponectin. Nuclear receptors, notably PPARγ, PPARδ, liver X receptor (LXR) and retinoid X receptor alpha (RXRα), integrate lipid metabolism with anti-inflammatory macrophage programming [20,21]. At the cytoskeletal level, lysophosphatidylserine (lyso-PS) activates G2A receptors, driving prostaglandin E2 (PGE2) synthesis; at nanomolar concentrations (≤1 nM), PGE2 enhances cAMP–PKA–Rac1 signaling, actin remodeling and efficient apoptotic cell uptake [22]. Mitochondrial regulators such as uncoupling protein 2 (UCP2) and a reduced mitochondrial membrane potential (ΔΨm) limit the generation of reactive oxygen species (ROS), thereby preventing the activation of the NLRP3 (NOD-like receptor family pyrin domain-containing 3) inflammasome and preserving Rac1 (Ras-related C3 botulinum toxin substrate 1)-dependent cytoskeletal dynamics essential for apoptotic cell clearance [19,23]. These stimulatory pathways collectively sustain physiological efferocytosis (Figure 2), ensuring efficient apoptotic cell clearance and restoration of tissue homeostasis.

By contrast, inhibitory pathways act at multiple control points. High mobility group box 1 (HMGB1), through binding to its cognate receptor for advanced glycation end-products (RAGE), together with Annexin A5, can conceal phosphatidylserine (PS) exposure or interfere with the activity of bridging molecules such as milk fat globule epidermal growth factor 8 (MFG-E8)—a glycoprotein that links PS on apoptotic cells to integrins on macrophages—thereby impairing apoptotic cell recognition and clearance [24,25]. At the intracellular signaling level, lysophosphatidic acid (LPA) activates RhoA (Ras homolog family member A), antagonizing Rac1 activity and reducing cytoskeletal rearrangements essential for apoptotic cell uptake [13]. Excessive mitochondrial ROS further promotes NLRP3 inflammasome activation, repressing Rac1 transcription and aggravating inflammatory signaling [23]. Importantly, PGE_2_ exhibits a biphasic effect: while nanomolar concentrations promote efferocytosis, higher concentrations (>10 nM) inhibit Rac1-dependent cytoskeletal remodeling and compromise apoptotic cell clearance [22]. These inhibitory mechanisms, some of which are illustrated in Figure 2, contribute to defective efferocytosis and the persistence of inflammation and tissue injury.

Collectively, these findings highlight the central role of the MerTK–PPARγ/LXR–Rac1 signaling axis in maintaining efficient efferocytosis. Disruption—via PS masking, RhoA activation, or ROS–NLRP3-mediated stress—shifts the system toward defective apoptotic cell clearance, thereby promoting persistent inflammation, tissue damage, and progression to chronic or neuroinflammatory pathology [13,23]. The dysregulation of efferocytosis becomes particularly apparent during viral infections, where defective clearance mechanisms perpetuate inflammation and neuroimmune activation [25].

At the molecular level, defective phagocytosis during viral infections reflects impaired receptor–ligand interactions and disrupted downstream signaling cascades [26]. In influenza A and SARS-CoV-2 infections, apoptotic cells normally externalize phosphatidylserine (PS), which is recognized by binding molecules such as MFG-E8 and Gas6, activating MerTK/Axl-mediated signaling [26,27,28]. This cascade recruits PI3K-Akt and ERK pathways, leading to PPARγ-dependent transcriptional programs that resolve inflammation [27,29]. Viral interference with PS externalization, Gas6/MFG-E8 binding, or MerTK/Axl receptor expression abrogates this anti-inflammatory reprogramming [30,31]. Consequently, apoptotic bodies accumulate and undergo secondary necrosis, releasing DAMPs, promoting NF-κB activation, and amplifying IL-1β, TNF-α, and IL-6 secretion [19,32]. In SARS-CoV-2, the cytokine storm further destabilizes phagocytosis, while mitochondrial ROS activates the NLRP3 inflammasome, inhibits Rac1-mediated cytoskeletal remodeling, and impairs the uptake of apoptotic cells [32].

Within the CNS, microglial phagocytosis is indispensable for the removal of apoptotic neurons, myelin fragments, and synaptic debris [26,33]. Microglia, the resident immune sentinels of the CNS, continuously monitor the neural milieu and detect chemotactic and phagocytic cues—such as the externalization of phosphatidylserine on apoptotic or injured neurons—that initiate the process of cellular clearance [26,34]. Impaired recognition or signaling via receptors including TREM2 (Triggering Receptor Expressed on Myeloid cells 2), MerTK or C1q compromises this clearance process, leading to the accumulation of neuronal debris and sustained activation of the NLRP3 inflammasome [26,33,35,36,37]. This dysfunction results in sustained release of pro-inflammatory cytokines, notably interleukin-1β (IL-1β) and interleukin-6 (IL-6), which drive microglial priming and chronic neuroinflammation [5,33,38].

This inflammatory milieu contributes to maladaptive nociceptor sensitization, characterized by increased release of excitatory neurotransmitters such as glutamate and ATP, upregulation of ion channels including Nav1.7 (voltage-gated sodium channel subtype 1.7) and TRPV1 (Transient Receptor Potential Vanilloid 1), and dysregulated glial–neuronal communication [33,39]. The resulting molecular and cellular alterations lower pain thresholds and promote persistent NP, mechanisms increasingly recognized in post-viral syndromes such as PASC [5,40,41]. Similar mechanisms are observed in neurodegenerative diseases including Alzheimer’s and Parkinson’s disease, where defective Gas6-Axl and TREM2-DAP12 (DNAX-activating protein of 12 kDa) signaling impedes efficient synaptic remodeling and clearance, exacerbating chronic neuroinflammation and neurodegeneration [42,43].

In cancer, efferocytosis exerts a context-dependent role. Tumor cells overexpress anti-phagocytic molecules such as the glycoprotein CD47, which engages SIRPα (Signal Regulatory Protein Alpha) on macrophages to inhibit phagocytic uptake and cytoskeletal rearrangement [44]. Conversely, efferocytosis of apoptotic tumor cells activates MerTK and Axl signaling pathways, resulting in STAT3 phosphorylation and increased production of IL-10 and TGF-β [44,45]. These signaling events promote macrophage polarization toward an M2 immunoregulatory phenotype that enhances angiogenesis, extracellular matrix remodeling, and ultimately drives tumor immune escape [45,46].

Taking together, dysregulated phagocytosis in viral infections, neurodegenerative disorders, and cancer represents a molecular control point linking defective clearance to systemic consequences. Aberrant regulation of the MerTK–PI3K–Akt–PPARγ, Gas6–Axl, NLRP3–ROS–Rac1, and CD47–SIRPα pathways not only perpetuates unresolved inflammation, but also establishes the molecular substrate for persistent NP, chronic neuroinflammation, and tumor progression [38,39,42,43,45,47]. Currently, therapies that activate resolution pathways show promise in chronic inflammatory and autoimmune diseases [1,3,7,48,49,50], although challenges remain due to mechanistic complexity, tissue-specific variability, and the lack of standardized biomarkers.

Accumulating experimental evidence highlights that defective efferocytosis is a central driver of unresolved neuroinflammation and NP. Kobayashi et al. (2020) [51] showed that peripheral nerve injury reduces MerTK expression in M2-polarized macrophages, impairing apoptotic cell clearance and promoting secondary necrosis with DAMP release, thereby sustaining NF-κB signaling and nociceptive hypersensitivity. Similarly, Zhao et al. (2023) [46] reported that insufficient efferocytic activity within the injured nerve microenvironment delays resolution and perpetuates chronic pain behaviors, while Kalinski et al. (2020) [52] demonstrated in a sciatic nerve injury model that efficient macrophage-mediated engulfment of apoptotic leukocytes fosters an anti-inflammatory milieu, attenuates cytokine release, and prevents persistent neuropathic hypersensitivity.

Additional insights from CNS models strengthen this concept. Soliman et al. (2023) [53] demonstrated that EphA4 (ephrin type-A receptor 4) signaling inhibits efferocytosis through ERK/STAT6/MerTK-dependent pathways, reducing apoptotic debris clearance in injured cortical tissue. This defective resolution response in the brain links impaired efferocytosis to sustained neuroinflammation, offering a mechanistic framework for post-viral or trauma-associated NP. At the systemic level, Wanke et al. (2021) [54] confirmed that MerTK kinase activity is essential for efferocytosis across murine and human models, reinforcing the idea that intact MerTK signaling represents a conserved molecular checkpoint for efficient apoptotic cell clearance.

Building upon these observations, Ruan et al. (2024) provided direct mechanistic evidence that enhancing efferocytosis alleviates NP [55]. In a chronic constriction injury (CCI) model [55], ozone therapy reduced mechanical hypersensitivity and neuroinflammation by activating the AMP-activated protein kinase (AMPK)/Gas6–MerTK signaling axis. Ozone increased AMPK phosphorylation and Gas6 expression, facilitated MerTK activation, and restored efferocytic capacity. In addition, it prevented A disintegrin and metalloproteinase 17 (ADAM17)-mediated MerTK cleavage, preserved receptor integrity, and enhanced apoptotic cell removal. This cascade increased suppressor of cytokine signaling 3c (SOCS3) protein level, which suppressed pro-inflammatory cytokines such as IL-1β, IL-6 and TNF-α. Pharmacological inhibition of AMPK or MerTK abrogated these effects, confirming the causal role of this pathway [55]. This preclinical evidence is complemented by the exploratory clinical study of Totolici et al. (2017) [56], which showed that ozone therapy, administered to palliative care patients, significantly improved antioxidant enzyme levels (superoxide dismutase and glutathione peroxidase), reduced pain perception, and enhanced quality of life—lending real-world translational relevance to the mechanistic findings of ozone-mediated activation of the AMPK/Gas6–MerTK pathway.

Conversely, therapeutic activation of pro-resolving pathways, particularly the AMPK/Gas6–MerTK/SOCS3 axis, restores resolution mechanisms and provides analgesic benefit, offering a promising framework for targeting post-viral and injury-induced neuroinflammatory states [55]. However, when these pro-resolving mechanisms fail to re-establish immune balance, macrophages and glial cells remain in a state of persistent activation, perpetuating inflammatory signaling and contributing to chronic neuroinflammation.

### 3.2. Persistent Activation of Macrophages and Glial Cells

Macrophages normally transition from a pro-inflammatory M1 phenotype, characterized by the production of inducible nitric oxide synthase (iNOS) and interleukin-1β (IL-1β) to an M2 reparative state determined by interleukin-10 (IL-10), transforming growth factor beta (TGF-β) and lipid mediators [2]. This shift, essential for tissue repair and inflammation resolution, depends on intact signaling through MerTK, STAT6, and PPARγ. When these pathways are disrupted, NF-κB and STAT1 transcriptional programs persist, maintaining a pro-inflammatory phenotype [57,58,59]. In the CNS, microglia and astrocytes display a parallel pattern of sustained activation, producing cytokines (IL-6, TNF-α) and reactive oxygen species (ROS) that drive chronic neuroinflammation and sensitize nociceptive pathways [58,60].

By releasing pro-inflammatory mediators, classically activated M1 macrophages act as early responders at inflammatory sites; however, during the resolution phase, they must transition to an anti-inflammatory M2 phenotype to eliminate apoptotic cells and facilitate tissue repair. Efferocytosis enables this shift by suppressing pro-inflammatory signaling and enhancing IL-10 and TGF-β release [2,61].

Beyond this classical description of macrophage polarization, efferocytosis has emerged as a central regulator of the M1-to-M2 transition. By clearing apoptotic cells, macrophages not only suppress inflammatory signaling, but also acquire metabolic substrates that sustain anti-inflammatory pathways programs. For instance, apoptotic cell-derived arginine and ornithine fuel continual efferocytosis and reinforce reparative functions, whereas fatty acid oxidation and glutaminolysis drive IL-10 synthesis and promote tissue remodeling [34,62,63]). These metabolic pathways illustrate the concept of “immunometabolic licensing,” whereby processing of apoptotic material generates the bioenergetic and biosynthetic capacity required to maintain a pro-resolving phenotype [62,63].

Efferocytosis also intersects with lipid mediator signaling. Prostaglandin E_2_ (PGE_2_), traditionally viewed as a pro-inflammatory mediator, plays a dual role: at low (nanomolar) concentrations, it enhances Rac1-dependent cytoskeletal remodeling and apoptotic cell uptake, whereas at higher concentrations, it inhibits efferocytosis and sustains inflammation [2,13,22,64].

When these mechanisms are compromised, resolution fails and macrophages remain locked in a pro-inflammatory M1-like state. Defective MerTK–PPARγ or STAT6 signaling, together with oxidative-stress-induced NLRP3 activation perpetuates IL-6, TNF-α and ROS release and reinforces a self-sustaining inflammatory loop [2,26,59,65]. In the CNS, this translates into impaired microglial and astrocytic clearance of apoptotic neurons and synaptic debris, persistent cytokine production, and activation of the inflammasome pathway [46,66].

These processes extend beyond unresolved inflammation to include maladaptive plasticity of pain pathways. Persistent glial activation lowers nociceptive thresholds via increased excitatory neurotransmitter release, upregulation of Nav1.7 and TRPV1 ion channels, and abnormal glial–neuronal crosstalk [33,39]. These molecular and cellular changes provide a mechanistic basis for chronic NP in post-viral syndromes (e.g., long COVID) and in neurodegenerative disorders where efferocytosis is impaired, including defective TREM2–DAP12 or Gas6–Axl signaling [5,42,43].

Experimental evidence converges on this model: impaired efferocytosis—whether due to MerTK downregulation, inhibitory receptor signaling (e.g., EphA4), or oxidative stress—leads to apoptotic cell accumulation, persistent macrophage/glial activation, and neuroinflammation [9,51,52,53,54]. Representative studies are summarized in Table 1. As detailed in Section 3.1, pro-resolving strategies that enhance efferocytosis—e.g., activation of the AMPK/Gas6–MerTK axis or limitating of ADAM17-mediated MerTK cleavage—can alleviate neuroinflammation and pain hypersensitivity [54,55,56].

Thus, macrophage and microglial reprogramming via efferocytosis and immunometabolic adaptation represent not only a determinant of inflammation resolution but also a molecular checkpoint in the prevention of chronic neuroinflammatory and neuropathic conditions [67,71].

### 3.3. Dysregulation of Specialized Pro-Resolving Mediators (SPMs)

**Figure 3:**
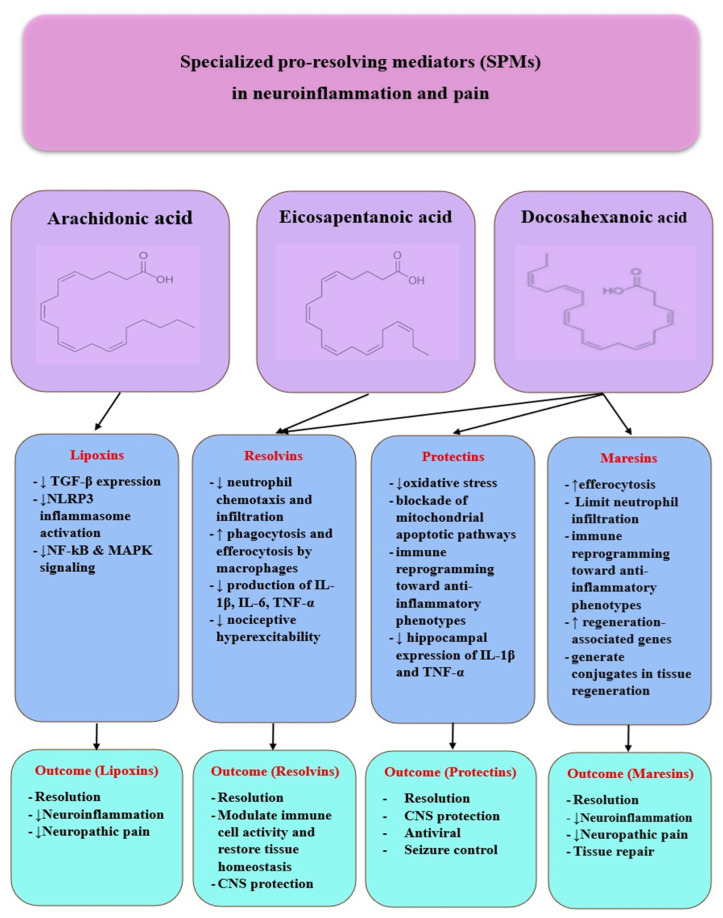
SPMs in neuroinflammation and pain. Specialized pro-resolving mediators (SPMs)—lipoxins, resolvins, protectins, and maresins—derived from AA, EPA, and DHA regulate neuroinflammation by downregulating pro-inflammatory pathways, modulating glial responses, and promoting neuronal protection and tissue repair, ultimately contributing to pain resolution.

Since persistent macrophage–glial activation reflects failed resolution and defective efferocytosis (Section 3.2), we next examine SPMs—lipoxins (LX), resolvins (Rv), protectins (PD), and maresins (MaR)—which are biosynthesized from arachidonic acid (AA), eicosapentaenoic acid (EPA), and docosahexaenoic acid (DHA) via lipoxygenase (LOX) and cyclooxygenase-2 (COX-2) pathways (Figure 3).

At the mechanistic level, SPMs act via G protein-coupled receptors such as Annexin A1/formyl peptide receptor 2 (ALX/FPR2), chemerin receptor 23 (ChemR23), and G protein-coupled receptor 32 (GPR32), thereby inhibiting neutrophil chemotaxis, enhancing efferocytosis and phagocytosis, and promoting tissue repair [1]. Impaired biosynthesis or receptor signaling diminishes SPM activity, shifting the balance toward persistent prostaglandin and leukotriene signaling and compromising macrophage reprogramming, thereby prolonging inflammation [72]. Beyond merely halting inflammation, SPMs actively orchestrate the resolution phase by coordinating interactions between neutrophils, macrophages, and epithelial cells, facilitating debris clearance and restoring tissue homeostasis [10,73,74,75].

#### 3.3.1. Lipoxins

Among SPM classes, lipoxins—arachidonate-derived mediators—provide prototypical evidence for resolution biology, with experimental studies demonstrating effects on inflammatory signaling, fibrogenesis, epithelial repair, and inflammatory tone [10,76].

In animal models of liver fibrosis, lipoxins downregulate TGF-β and α-SMA expression and prevent excessive collagen deposition [77]. In genetic models, the absence of LOX enzymes (Alox5, Alox15) leads to persistent inflammation and elevated pro-inflammatory cytokines [78,79]. In an ex vivo study of nasal epithelium from patients with cystic fibrosis, treatment with RvE1 and LXB4 (10 nM) restored ciliary beating dynamics, airway surface liquid, and reduced mucus thickness, demonstrating a direct epithelial-restorative effect [80].

In preclinical models, LXA4 has also been shown to inhibit NLRP3 inflammasome activation, to suppress NF-κB and MAPK signaling and to reduce the expression of pro-inflammatory mediators such as TNF-α, IL-1β, and IL-6 [10,77,81]. Moreover, treatment with stable LXA4 analogs exerted protective effects in acute lung injury models by limiting neutrophil infiltration, alleviating alveolar damage, and enhancing alveolar fluid clearance [77,82].

Clinical data provide complementary insights. An observational study in critically ill COVID-19 patients admitted to the ICU [83] showed that serum levels of LXA4 did not differ significantly between patients (regardless of disease severity) and healthy controls, suggesting a possible inability of the host to mount an effective pro-resolving response. The authors hypothesized that the lack of a “PGE2 peak” required for LXA4 induction could explain this deficit. In contrast, other specialized pro-resolving mediators (RvE1, MaR2, and RvD5) were elevated in patients with severe disease, indicating that pro-resolving mechanisms are activated, but insufficient to counteract inflammation.

Complementary results were reported by Sánchez-García et al. [81], where low serum LXA4 levels were inversely correlated with SOFA scores and mortality in COVID-19 patients. The deficit was attributed to reduced expression of the 15-LOX enzyme and accelerated degradation via 15-PGDH. Moreover, LXA4 showed negative correlations with tissue injury markers (CRP, LDH, D-dimer, and ferritin) and pro-inflammatory cytokines (IL-6, IL-8, CCL2, and CXCL10). A serum threshold of 124.1 pg/mL was proposed as a prognostic discriminator, with high sensitivity and specificity [81].

Taken together, these experimental and clinical findings reinforce the central role of LXA4 as a regulator of inflammation and resolution. They suggest that insufficient endogenous production during viral infections, such as COVID-19, contributes to disease severity, while preclinical models consistently demonstrate its protective actions. This dual perspective positions LXA4 not only as a biomarker of severity, but also as a promising therapeutic target using stable analogs or strategies to enhance its biosynthesis, in line with the broader ability of SPMs to inhibit NF-κB and MAPK signaling pathways, modulate the NLRP3 inflammasome, and reduce oxidative stress and pro-inflammatory cytokine production.

Furthermore, Lipoxin A4 (LXA4) has been increasingly recognized as a key regulator of neuroinflammation. In experimental models, administration of LXA4 reduced glial activation, suppressed TNF-α and other pro-inflammatory mediators and alleviated NP [76]. Similar protective effects were observed when stimulation of ALX/FPR2 signaling attenuated microglial reactivity in intracerebral hemorrhage [4]. In neurodegeneration, a nanostructured LXA4 formulation inhibited microglial activation, reduced pro-inflammatory cytokines, enhanced β-amyloid clearance, and preserved cognitive performance [84]. Aspirin-triggered LXA4 also attenuated hyperalgesia and anxiety-like behavior in diabetic neuropathy, with enhanced antinociceptive effects in combination with cannabinoid receptor agonists [85]. These findings gain further relevance in the context of post-viral syndromes, where unresolved neuroinflammation—through NLRP3 inflammasome activation, cytokine dysregulation, and blood–brain barrier disruption—has been highlighted as a driver of cognitive impairment in Long COVID [5]. Moreover, studies in SARS-CoV-2 infection models have shown that LXB4 can enhance B-cell responses by prolonging plasma-cell survival and anti-apoptotic B-cell lymphoma -2 (BCL-2) family proteins, potentially improving vaccine responsiveness [86,87,88]. In addition, LXA4 alleviates neuroinflammation across multiple models (multiple sclerosis, Alzheimer’s disease, Parkinson’s disease, and cerebral ischemia) by modulating T-cell activity, activating PPARγ, and interacting with the endocannabinoid system, while intracerebroventricular administration in stroke models further improved neurological outcomes and reduced infarct volume [67,88,89]. Collectively, this evidence positions LXA4 as a promising therapeutic target not only for systemic and pulmonary inflammation, but also for NP, neurodegeneration, and post-COVID neurocognitive sequelae [8].

#### 3.3.2. Resolvins

Resolvins belong to the family of SPMs, together with lipoxins, protectins, and maresins [1]. They are derived from omega-3 poly-unsaturated fatty acids, particularly eicosapentaenoic acid (EPA), the precursor for E-series resolvins and docosahexaenoic acid (DHA), the precursor for D-series resolvins. Their biosynthesis is mediated by specific lipoxygenases (5-LOX, 12-LOX, and 15-LOX) and, under certain conditions, by aspirin-acetylated cyclooxygenase-2 (COX-2), which generates aspirin-triggered (AT) resolvins [1,83]. These molecules act through G protein-coupled receptors such as ChemR23, ALX/FPR2, and GPR32, thereby modulating immune cell activity and restoring tissue homeostasis [1].

Resolvins are not merely inhibitors of inflammation, but active orchestrators of the resolution phase [1]. They suppress neutrophil chemotaxis and tissue infiltration, enhance macrophage-mediated phagocytosis and efferocytosis, limit the production of pro-inflammatory cytokines (IL-1β, IL-6, and TNF-α), and promote macrophage polarization toward the pro-repair M2 phenotype. Through these coordinated actions, resolvins prevent the transition from acute to chronic inflammation and facilitate tissue repair and regeneration [3,90,91].

In the context of COVID-19, several studies have reported marked disturbances in circulating lipid mediator profiles, including resolvins [83,92,93]. Patients with severe COVID-19 exhibited increased plasma concentrations of RvE1, MaR2, and RvD5ș; however, these elevations remained insufficient to counterbalance the hyperinflammatory cytokine storm, whereas LXA4 levels remained consistently low. Defective biosynthesis or enhanced degradation of SPMs, including resolvins, correlated with disease severity and with elevated markers of tissue injury such as CRP, LDH, D-dimer, and ferritin [81,83]. These findings suggest that in COVID-19, pro-resolving pathways are only partially activated and fail to restore inflammatory homeostasis, thereby contributing to persistent inflammation. Consequently, resolvins have been proposed as promising biomarkers of disease severity and as potential therapeutic targets [2,8].

The deficiency or functional impairment of resolvins also has major consequences in CNS. Reduced SPM levels sustain microglial and astrocytic activation, amplify NLRP3 inflammasome activity, and promote persistent secretion of IL-1β, IL-6, and TNF-α [94]. This chronic pro-inflammatory milieu contributes to blood–brain barrier (BBB) dysfunction, neuroinflammation, and nociceptive sensitization, all of which are essential mechanisms underlying NP and cognitive symptoms observed in long COVID [5]. Experimental studies have shown that resolvins attenuate microglial activation, suppress pro-inflammatory signaling, and enhance the clearance of neuronal debris, thereby limiting neurodegeneration and cognitive impairment [76,84]. By activating ALX/FPR2 and ChemR23 receptors, resolvins modulate glia–neuron crosstalk and reduce neuronal hyperexcitability, providing neuroprotective effects in post-viral neuropathy and COVID-19–associated neuroinflammation [4,85].

Therapeutic applications of resolvins have demonstrated potential in both oncology and neurology. Resolvin D2 (RvD2) has been shown to attenuate chronic neuropathic and bone cancer pain by inhibiting spinal IL-17 secretion, CXCL1 release, and astrocyte activation [95], whereas Resolvin D1 (RvD1) facilitates the resolution of neuroinflammation by suppressing microglial activation through the BDNF/TrkB signaling pathway [73]. More recently, a new subclass of pro-resolving mediators, the T-series resolvins (RvT1–RvT4), have been identified. These molecules reduce excessive neutrophil extracellular trap (NET) formation and promote macrophage-mediated NET clearance via a cAMP–PKA–AMPK axis [96]. Such mechanisms are particularly relevant in COVID-19, where dysregulated NETosis and impaired resolution contribute to systemic inflammation, coagulopathy, and potentially to neuroinflammation and long-term sequelae [90,96,97].

#### 3.3.3. Protectins

Protectins (PD), derived from docosahexaenoic acid (DHA), are SPMs involved in the resolution of inflammation and tissue protection through mechanisms that include the inhibition of oxidative stress, blockade of mitochondrial apoptotic pathways, and immune reprogramming toward anti-inflammatory phenotypes [9]. Neuroprotectin D1 (NPD1), the best-characterized member of this class, has been shown to attenuate excitotoxicity, oxidative stress and glial activation, while preserving neuronal structure and function [9]. In experimental models of cerebral ischemia, NPD1 administration reduced infarct volume and improved neurological recovery through the inhibition of mitochondria-dependent apoptotic pathways, induction of the DNA-repair protein Iduna and preservation of BBB integrity [9]. Furthermore, NPD1 promotes neurogenesis and angiogenesis, consolidating its role in regenerative processes and underscoring the association between reduced protectin levels and the progression of neurodegenerative disorders, including Alzheimer’s disease [9]. Synthetic analogs further extend this therapeutic potential: the compound 3-oxa-PD1n-3 DPA exhibited superior antiallodynic and antipruritic activity compared with native PD1 in murine models of diabetic neuropathy and chronic itch [98].

The role of protectins also encompasses antiviral responses and immune balance regulation [9]. In respiratory syncytial virus (RSV) infection, protectin conjugates in tissue regeneration 1 (PCTR1) and PD1 reduced neutrophil and eosinophil infiltration, restored interferon-λ expression, and induced the antimicrobial peptide cathelicidin (LL-37), thereby limiting viral replication and lung inflammation without inducing immunosuppression [99]. In COVID-19, elevated plasma PD1 levels were detected in critically ill patients and were associated with macrophage polarization toward the anti-inflammatory M2 phenotype and increased IL-10 production, supporting the resolution of hyperinflammation [100]. Complementary evidence indicates that bioactive lipids—including protectins, resolvins, and maresins—facilitate viral clearance, modulate vascular responses, and exert antiviral effects against enveloped viruses [101].

Moreover, protectin DX (PDX) analogs provide new therapeutic perspectives. Fortin et al. (2024) [102] demonstrated that the synthetic analog AN-137B inhibited replication of influenza A(H1N1) viruses, including oseltamivir- and baloxavir-resistant strains, in a dose-dependent manner with a favorable selectivity profile. In LPS-stimulated macrophages, AN-137B reduced inducible nitric oxide synthase (iNOS) activity and nitrite production, confirming a direct anti-inflammatory action. Importantly, combinations with classical antivirals showed synergistic effects with oseltamivir and additive effects with baloxavir, highlighting the value of multimodal therapy that simultaneously targets viral replication and inflammatory cascades [102].

Thus, protectins and their analogs act through a complex network of mechanisms—including the regulation of mitochondrial survival pathways, modulation of interferon responses, induction of antimicrobial peptides, macrophage reprogramming, and control of oxidative stress—placing them among the most promising therapeutic candidates for managing acute viral infections and preventing chronic post-viral complications, including neuroinflammation and cognitive dysfunction associated with PASC.

Beyond their antiviral actions, protectins are increasingly recognized as key regulators of neuroinflammatory responses. In epilepsy models, the n-3 docosapentaenoic acid (DPA)–derived protectin D1 (PD1n-3 DPA) suppressed hippocampal expression of IL-1β and TNF-α, restored lipid mediator balance and improved cognitive performance. Intracerebroventricular administration of PD1n-3 DPA significantly reduced both the frequency and duration of spontaneous seizures, demonstrating that insufficient engagement of resolution pathways sustains neuronal hyperexcitability. Pro-resolving receptors such as ALX/FPR2 and ChemR23 were also found upregulated in astrocytes from epileptogenic tissue of both mice and patients with temporal lobe epilepsy, underscoring the translational relevance of these mechanisms [103].

Protectins also operate in concert with other families of SPMs, including resolvins and maresins, to coordinate the resolution of neuroinflammation. Resolvins inhibit microglial activation, reduce pro-inflammatory cytokine release, and attenuate pain hypersensitivity, while maresins promote macrophage polarization, efferocytosis, and tissue repair [1]. Together, these mediators form an integrated network in which protectins stabilize mitochondrial function and limit apoptosis, resolvins counteract immune-driven excitotoxicity, and maresins support regeneration and homeostasis [103].

Altogether, this evidence positions protectins, alongside resolvins and maresins, as crucial mediators at the intersection of neuroprotection, inflammation resolution and seizure control, providing a strong mechanistic rationale for their therapeutic exploration in chronic neuroinflammatory and neurodegenerative disorders.

#### 3.3.4. Maresins

Maresins—another family of SPMs from DHA—play a pivotal role in inflammation resolution, tissue regeneration and immune homeostasis [74]. They are biosynthesized in macrophages through the 12-LOX pathway and act through G-protein-coupled receptors such as leucine-rich repeat-containing G protein-coupled receptor 6 (LGR6) and ALX/FPR2 to enhance efferocytosis, limit neutrophil infiltration, and promote a switch toward pro-resolutive (M2-like) macrophage phenotypes. In addition to dampening excessive cytokine release, maresins stimulate tissue repair programs by inducing regeneration-associated genes and by generating maresin conjugates in tissue regeneration (MCTR), which couple infection clearance with repair mechanisms [74,104].

Clinical evidence now extends these findings to PASC. In a 12-week interventional study [92], supplementation with marine oil enriched in SPM precursors—including resolvins, protectins, and maresins—led to a significant increase in circulating 14-hydroxydocosahexaenoic acid (14-HDHA), 17-HDHA, and 18-hydroxyeicosapentaenoic acid (18-HEPE), together with a shift in the ratio of pro-inflammatory versus pro-resolutive lipid mediators. These biochemical changes correlated with improvements in fatigue and dyspnea, two hallmark PASC symptoms, underscoring the translational potential of maresins and related SPMs in counteracting persistent inflammation and promoting recovery in post-viral syndromes [105,106].

Maresins, particularly Maresin-1 (MaR1), have emerged as potent regulators of neuroinflammation and NP [74,107]. In surgical models, systemic or intracerebral administration of MaR1 attenuated trauma-induced hippocampal inflammation, reduced astrocyte reactivity, and preserved cognitive performance, underscoring its ability to counteract perioperative neurocognitive decline [106]. In peripheral nerve injury models, MaR1 promoted axonal regeneration, suppressed spinal microglial and astrocytic activation, and alleviated mechanical allodynia and thermal hyperalgesia, effects mediated via inhibition of TRPV1 currents and modulation of the PI3K–AKT–mTOR signaling pathway [107]. Complementary findings in inflammatory pain demonstrated that MaR1 reduced NF-κB activation, cytokine release (IL-1β, TNF-α), and calcitonin gene-related peptide (CGRP) secretion by dorsal root ganglion neurons, resulting in long-lasting analgesic effects even at nanogram doses [71].

Notably, Maresin-2 (MaR2) has also been shown to accelerate tissue repair and enhance resolution of inflammation, with overlapping, but distinct actions from MaR1 that may be particularly relevant in chronic pain states and central neuroinflammation [108]. Experimental evidence indicates that intrathecal administration of MaR2 reduces orofacial nociceptive behavior, prevents postoperative hyperalgesia, and reverses trigeminal NP by suppressing c-Fos activation and NF-κB+/CGRP+ neurons in the trigeminal ganglion, thereby restoring neuroimmune homeostasis [108].

These mechanistic insights suggest that maresins may be especially valuable in the context of chronic NP, where persistent neuroinflammation, maladaptive glial activation, and dysregulated nociceptive signaling maintain hypersensitivity long after the initial insult [71,105,106,107,108]. By reprogramming macrophages toward pro-resolving phenotypes and enhancing efferocytosis, maresins could also counteract the sustained immune activation observed after viral infections, including herpesviruses and SARS-CoV-2, which are known to trigger or exacerbate chronic pain syndromes [1,108].

In this broader framework, maresins act synergistically with other SPMs—including protectins and resolvins—to simultaneously limit excessive inflammation, promote tissue repair, and restore neuronal circuit stability [103]. This positions maresins as promising therapeutic candidates for targeting the intersection between chronic neuroinflammation, post-viral sequelae, and persistent NP.

In summary, SPMs constitute an integrated network of lipid-derived molecules that promote the termination of inflammation, restoration of tissue homeostasis, and neuronal protection. By activating receptors such as ALX/FPR2, ChemR23, GPR32, and LGR6, SPMs modulate immune and glial responses, suppress inflammasome activation, and support tissue repair processes. Their key l characteristics, mechanistic roles, and clinical implications are summarized in Table 2.

### 3.4. Mitochondrial Dysfunction

Mitochondria integrate inflammatory and metabolic signals [60]. Viral infections and prolonged cytokine exposure disrupt oxidative phosphorylation and increase mitochondrial reactive oxygen species (mtROS production) [111]. Elevated mtROS activate inflammasomes, such as NLRP3, amplifying the release of interleukin-1β (IL-1β) and interleukin-18 (IL-18) [5], while impaired mitophagy allows the accumulation of dysfunctional mitochondria [2]. These events inhibit efferocytosis, maintain persistent NF-κB activation and disrupt metabolic coupling between neurons and glia [60]. The result is a feed-forward loop of bioenergetic failure, unresolved inflammation, and neuroinflammatory sensitization that underlie post-viral NP [111].

Evidence of mitochondrial involvement in long COVID comes from biomarker studies showing a systemic redox imbalance: circulating markers of lipid peroxidation (e.g., F2-isoprostanes, malondialdehyde) are elevated, while endogenous antioxidants such as coenzyme Q10 are reduced [58,60,111,112]. In parallel, transcriptomic analyses [113,114] reveal differential expression of genes governing mitochondrial metabolism, mitophagy and antiviral defense, consistent with impaired ATP production and excess ROS. This bioenergetic stress correlates closely with the clinical phenotype—fatigue, exertional intolerance, myalgias, and cognitive dysfunction—and helps explain the persistence of symptoms despite resolution of the acute infection [111].

Comparable patterns are seen in other post-infectious conditions. In myalgic encephalomyelitis/chronic fatigue syndrome (ME/CFS), studies document respiratory-chain dysfunction, diminished ATP generation, elevated lactate, and increased oxidative stress, closely resembling long-COVID signatures [65,115,116]. Post-treatment Lyme disease [117] and chronic sequelae after herpesvirus infections [118] likewise implicate mitochondrial fragmentation or sustained immune activation as drivers of persistent energetic failure, while Q fever fatigue syndrome presents a similar constellation of symptoms suggestive of overlapping mechanisms [119].

Together, these lines of evidence converge on a shared pathway—infection-induced mitochondrial injury leading to energy deficit and oxidative stress—highlighting mitochondrial support and redox-targeted strategies as rational therapeutic avenues for long COVID and related post-infectious syndromes [111].

**Figure 4:**
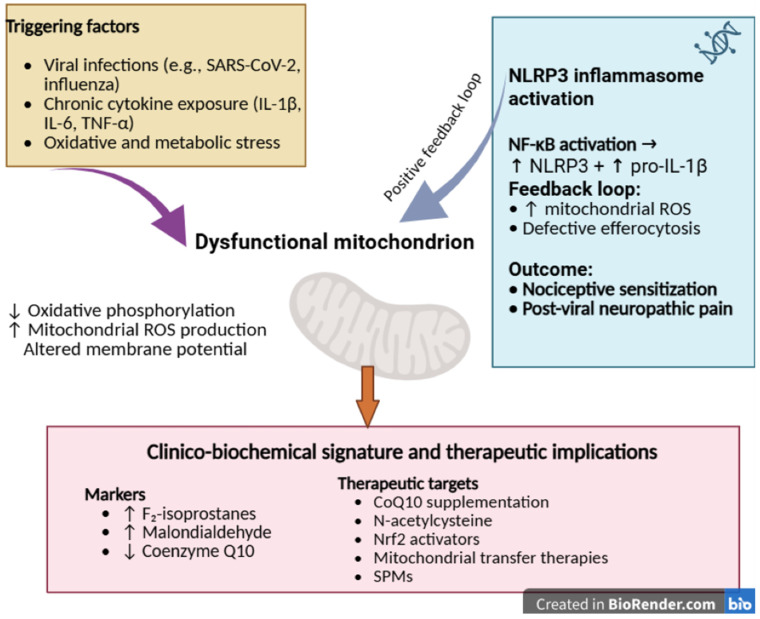
Mechanisms of mitochondrial dysfunction in post-viral inflammation and neuropathic pain.

Figure 4 summarizes the proposed mechanisms linking mitochondrial dysfunction to neuroinflammation and post-viral NP. Disruption of oxidative phosphorylation and impaired mitochondrial metabolism lead to mtROS accumulation, NLRP3 activation, and sustained NF-κB signaling, thereby driving glial activation and nociceptive sensitization that contribute to long-term neurological sequelae.

Viral infections, chronic cytokine exposure, and oxidative stress impair mitochondrial respiration and metabolic coupling, leading to increased mtROS production, altered membrane potential (ΔΨm), and activation of the NLRP3 inflammasome and NF-κB signaling. These processes inhibit efferocytosis and perpetuate neuroinflammation, resulting in nociceptive hypersensitivity and chronic post-viral pain. Potential therapeutic targets include Nrf2 activators, CoQ10, N-acetylcysteine (NAC), mitochondrial transfer, and SPMs.

The mechanisms outlined above—from defective efferocytosis and persistent glial activation to oxidative and mitochondrial stress—illustrate a biological continuum of dysregulated inflammation following viral infection. Beyond the cellular and molecular levels, this state emerges within a broader neuroimmune–metabolic network that integrates systemic and psychosocial influences. Chronic inflammation and mitochondrial dysfunction can interact bidirectionally with psychological stress through altered neuroenergetics, hypothalamic–pituitary–adrenal (HPA) axis activation, and redox imbalance, creating a self-perpetuating feedback loop that delays resolution [5,65,87,113]. In this context, psychosocial stress and impaired mental health may amplify inflammatory persistence and increase vulnerability to post-viral NP. A web-based study conducted among Romanian adults during the COVID-19 pandemic [120] reported increased anxiety, depression, and perceived stress, underscoring the interplay between psychological burden and systemic inflammation and its potential contribution to post-viral neuroinflammatory syndromes.

### 3.5. Summary of Key Mechanistic Insights

The mechanistic evidence synthesized in this review indicates that respiratory viral infections can initiate a cascade of dysregulated inflammatory processes that fail to transition into a pro-resolving state. Across experimental and clinical studies, three convergent pathways emerge as central drivers of post-viral neuroinflammation and NP: (i) sustained activation of pro-inflammatory signaling networks, including epithelial–immune crosstalk, cytokine amplification, and inflammasome activation; (ii) oxidative–mitochondrial stress and metabolic reprogramming in immune and neural cells, which reinforce maladaptive innate immune activity; and (iii) impaired efferocytosis and deficient biosynthesis or signaling of SPMs, leading to prolonged microglial priming and neuroimmune dysregulation. Together, these interconnected processes drive the transition from acute viral inflammation to chronic neuroinflammatory and neuropathic states.

## 4. Discussion

### 4.1. Integration of Mechanisms with Other Chronic Inflammatory Diseases

Post-viral neuropathic pain represents a paradigmatic example of disrupted inflammatory resolution following respiratory viral infection, illustrating the transition from a protective acute inflammatory response to a maladaptive chronic state [66]. The persistence of the neuroinflammatory microenvironment is sustained by an interconnected mechanism: inefficient efferocytosis and impaired clearance of apoptotic cells and tissue debris; continuous activation of macrophages, microglia, and astrocytes; reduced production of SPMs, required for terminating inflammation; and altered mitochondrial homeostasis, leading to excessive ROS generation and amplification of inflammatory stress [19,26,121]. These dysregulated processes sensitize central and peripheral nociceptive circuits and promote the development and maintenance of chronic NP [see Table 2].

A similar same pathogenic pattern—defined by failure to efficiently resolve inflammation and restore immune-neuronal homeostasis—is observed across multiple chronic inflammatory disorders. In neurodegeneration, such as Alzheimer’s disease [122], microglia persist in a pro-inflammatory phenotype characterized by sustained IL-1β, TNF-α, and ROS secretion, while deficiencies in TREM2 and Gas6–Axl signaling impair the clearance of β-amyloid and synaptic debris, thereby perpetuating neuroinflammation and cognitive decline [36,123]. Wanke et al. (2021) [54] demonstrated that MERTK kinase activity is indispensable for efferocytosis in both murine and human macrophages, underscoring its conserved role as a molecular checkpoint for inflammation resolution. Defective of MERTK signaling leads to the accumulation of apoptotic cell, secondary necrosis, and amplification of pro-inflammatory cascades.

Comparable alterations are present in autoimmune diseases. In systemic lupus erythematosus, defective apoptotic clearance results to persistent exposure to nuclear autoantigens, activation of the adaptive immune system, and chronic systemic inflammation [124]. In rheumatoid arthritis, synoviocytes and macrophages maintain a chronic pro-inflammatory profile, sustained by IL-6 and TNF-α, inhibiting repair and promoting joint destruction [125]. Marchand et al. (2023) [125] reported that RA patients display altered circulating profiles of SPM precursors, while fish-oil supplementation increases EPA- and DHA-derived SPM intermediates, suggesting that endogenous resolution pathways are impaired, but can be pharmacologically restored. Similarly, in osteoarthritis, impaired mitochondrial homeostasis and oxidative stress contributes to persistence of pain, whereas dimethyl fumarate improves mitochondrial biogenesis via Nrf2 activation and alleviated pain behaviors in experimental models [126]. Multiple sclerosis also features defective efferocytosis of degenerated myelin and NLRP3 inflammasome activation, sustaining CNS inflammation, contributing to progressive demyelination and neurodegeneration. Lipidomic analyses confirm that MS patients exhibit reduced levels of pro-resolving mediators (LXA_4_, RvD_1_, and PD_1_), correlating with disease severity, indicating systemic failure of resolution programs [127].

Importantly, beyond innate immunity, SPMs also regulate adaptive immune response. They promote macrophage polarization toward a pro-resolving M2 phenotype, restore efferocytosis, and limit inflammasome activation. In parallel, they regulate B-cell maturation and antibody production, acting as immune adjuvants or suppressors depending on the immunological context [124]. Such evidence reinforces the concept that impaired resolution is not only a feature of local tissue inflammation, but reflects a systemic immunoregulatory dysfunction shared across chronic diseases [124,127,128].

Therefore, the mechanisms described in post-viral NP should not be regarded as isolated phenomena, but rather as part of a broader spectrum of shared inflammatory dysregulation. This convergence underscores unresolved inflammation as a central pathogenic hub linking post-viral syndromes with chronic inflammatory disorders. Crucially, these parallels open translational therapeutic opportunities, suggesting that strategies aimed at restoring inflammation resolution—by enhancing efferocytosis, modulating SPM signaling, or targeting redox homeostasis—may hold promise across multiple disease contexts [8,75,124,127].

### 4.2. Therapeutic and Clinical Perspectives

Recognizing the pathogenic overlaps between post-viral NP and other chronic inflammatory diseases opens new and clinically significant avenues for developing therapies that specifically target the resolution of inflammation [8,129]. The concept of resolution pharmacology is gaining increasing relevance, aiming not only to suppress inflammation but also to actively restore immune and tissue homeostasis by stimulating endogenous resolution mechanisms [8]. In particular, the use of analogs of SPMs—including lipoxins, resolvins, protectins, and maresins—holds promise for correcting the endogenous pro-resolving deficit observed in post-viral conditions and for attenuating nociceptive sensitization [73,82,99,108,129]. Likewise, activation of the MerTK/Gas6–Axl signaling axis represent an innovative strategy to restore effective efferocytosis and accelerate the transition toward a tolerogenic and reparative immune state. Experimental evidence demonstrates that MerTK kinase activity is indispensable for apoptotic cell clearance and its stimulation not only enhances efferocytosis, but also promotes a phenotypic switch of macrophages and microglia toward an anti-inflammatory M2 profile, increasing IL-10 while suppressing pro-inflammatory cytokines, such as IL-1β and TNF-α [54]. These effects are particularly relevant in post-viral NP, where sustained glial activation drives nociceptive sensitization, suggesting that MerTK/Gas6–Axl-based interventions could represent a viable and targeted therapeutic strategy [5,54].

Modulation of mitochondrial function has also emerged as a promising therapeutic avenue, as it is essential for reducing excessive ROS production and restoring neuronal energy balance, with direct implications for microglial reactivity and nociceptor excitability in NP [126]. Recent experimental work has highlighted that enhancing intercellular mitochondrial transfer, particularly via mesenchymal stem cells (MSCs), can restore bioenergetics, reduce oxidative stress and reprogram the immune microenvironment, ultimately attenuating pain behaviors in discogenic and inflammatory pain models [130].

In parallel, combined therapeutic strategies that integrate immunomodulation with metabolic support may provide synergistic benefits. For example, experimental studies have demonstrated that lipid mediators, such as MaR1 and RvD5, can suppress microglial activation, limit oxidative stress, and attenuate both neuropathic and inflammatory pain in preclinical models [107,129,131]. Moreover, the exploration of pro-resolving biomarkers has emerged as a valuable translational approach: reduced circulating levels of lipid mediators, such as LXA_4_, RvD_1_, and PD_1_, have been experimentally detected in patients with multiple sclerosis and are shown to correlate with disease activity, while supplementation with omega-3 fatty acids in rheumatoid arthritis patients restored circulating SPM profiles and promoted resolution pathways [73,125,127].

Taken together, shifting research efforts toward restoring the resolution of inflammation—rather than relying solely on nonspecific suppression of the immune response—has the potential to redefine the therapeutic approach to post-viral NP. Furthermore, these strategies carry significant translational relevance, as they may also be applicable to other disorders characterized by unresolved inflammation, spanning from neurodegenerative to autoimmune diseases. In this context, post-viral NP emerges not only as a complication of respiratory viral infections, but also as a pathophysiological model that can guide the development of innovative, resolution-oriented therapies with broad clinical applicability.

**Figure 5:**
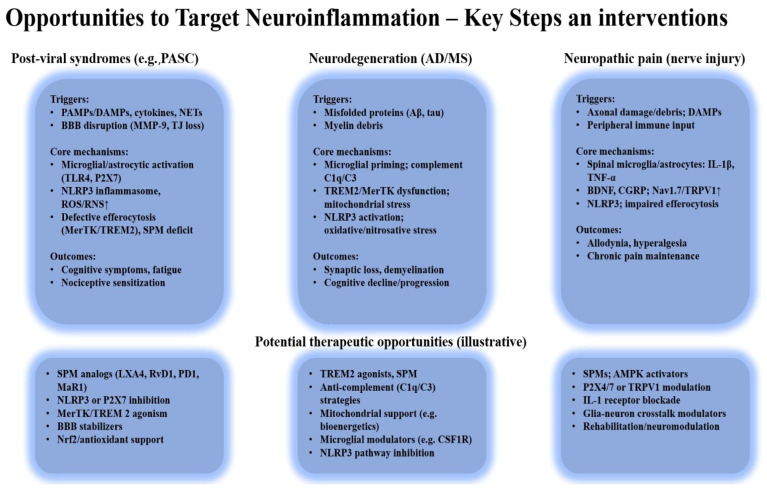
Opportunities to target neuroinflammation: key mechanisms and therapeutic intervention.

Figure 5 summarizes these interrelated mechanisms and highlights the main therapeutic targets involved in restoring immune homeostasis and promoting inflammation resolution.

Shared mechanisms—such as defective efferocytosis, NLRP3 activation, and mitochondrial dysfunction—drive persistent neuroinflammation and nociceptive sensitization. Therapeutic targets include SPMs, MerTK/TREM2 signaling, mitochondrial support, and immunometabolic modulation to restore homeostasis.

### 4.3. Prevention Through Vaccination: Insights into Neuroinflammation and Pathways to Resolution

Vaccination provides a controlled model to study the dynamics of immune activation and resolution, offering important insights into how transient inflammatory responses are contained and homeostasis is restored [132].

SARS-CoV-2 mRNA and vector-based vaccines transiently activate innate immune sensors such as Toll-like receptors (TLR7/8) and NF-κB, inducing short-lived cytokine elevations (IL-6, TNF-α, and IFN-γ) that promote adaptive immunity while remaining self-limited and physiologically regulated [133,134,135].

Large-scale cohort and registry studies consistently confirm the excellent safety profile of COVID-19 vaccines, with only rare and generally reversible cases of CNS inflammation temporally associated with immunization. Such events are reported primarily in individuals with pre-existing autoimmune predisposition or recent infection [136,137,138,139,140,141]. The absolute risk of vaccine-associated neuroinflammation remains exceedingly low and most cases resolve spontaneously or respond favorably to conventional anti-inflammatory therapy [136,137,138,139,140,141]. Beyond their preventive role against infection-induced neuroinflammation, vaccination also serves as a translational framework to explore how effective immune priming transitions into timely resolution—a dynamic equilibrium essential for maintaining neural homeostasis and long-term neuroprotection [132,142,143,144].

These observations highlight the need for integrated biomarkers capable of distinguishing physiological immune activation from maladaptive persistence, supporting the conceptual foundation of the proposed Resolution Failure Index (RFI) in the following section.

### 4.4. Potential for Predictive Algorithms and Personalized Approaches

Recent advances in computational biology and clinical data integration open new perspectives for developing predictive algorithms capable of identifying individuals at risk of persistent inflammation after viral infection [87,145]. Such tools could combine clinical parameters (e.g., duration and severity of acute infection, comorbidities, and immune markers) with molecular and lipidomic profiles—including cytokines, oxidative stress markers, and SPMs—to estimate the likelihood of transition from acute to chronic neuroinflammation.

Machine learning models [146] trained on these multidimensional datasets may enable the early detection of patients prone to unresolved inflammation and guide precision-tailored interventions aimed at restoring immune resolution. Integrating such algorithms into clinical practice could enhance patient stratification, improve follow-up, and accelerate the translation of resolution-based therapies from bench to bedside.

## 5. Predictive Frameworks and the Conceptual Basis for a Resolution Failure Index (RFI)

Growing efforts in computational and molecular profiling have sought to predict post-infectious sequelae, including PASC and post-viral NP, through multi-omics integration and inflammatory signature analysis.

Current machine learning models, based on electronic health records (EHRs) and incorporating circulating cytokines (IL-6, IL-1β, and TNF-α) alongside clinical and laboratory data, show moderate predictive accuracy for post-acute sequelae, with reported AUC values between 0.71 and 0.85, yet they remain heterogeneous and mechanistically limited [19,61,147]. Similarly, lipidomic signatures expressing the ratio between SPMs and pro-inflammatory cytokines offer valuable insight into the imbalance between inflammation and resolution [1,125], although these metrics are only partially validated and rarely integrated with oxidative or mitochondrial parameters. Other studies have explored mitochondrial and oxidative stress biomarkers—such as F_2_-isoprostanes, circulating cell-free mitochondrial DNA (cf-mtDNA), and lactate/pyruvate ratio—as indices of bioenergetic dysfunction [5,9,130], while neuroinflammatory panels (NLRP3, P2 × 7, GFAP, NfL) have been correlated with glial activation and pain sensitization [5,51,55].

Despite these advances, no integrative metric currently exists to quantify the failure of inflammatory resolution—a process linking defective efferocytosis, reduced SPM biosynthesis, mitochondrial dysfunction, and glial activation—with clinical outcomes such as NP persistence.

To address this gap, we propose the Resolution Failure Index (RFI) as a multidimensional framework integrating three interdependent biological dimensions that capture the persistence of neuroinflammation:

(i) A pro-inflammatory dimension, represented by cytokines such as IL-6, TNF-α, IL-1β, and hs-CRP [5,19],
(ii) A pro-resolving deficit dimension, reflecting reduced levels of SPMs—including LXA4, RvD1, PD1, and MaR1 [121]; and
(iii) An oxidative–mitochondrial stress dimension [111], comprising biomarkers such as F_2_-isoprostanes, malondialdehyde (MDA), cf-mtDNA, lactate/pyruvate ratio, and CoQ10.

Together, these dimensions capture the biological continuum linking sustained inflammation, oxidative stress, and impaired resolution, ultimately promoting nociceptive sensitization and chronic pain [8].

At a conceptual level, each biomarker within these axes could be standardized using Z-scores [148], enabling the construction of composite indices mathematically integrating the balance between inflammatory activation and resolution capacity.

The RFI thus represents a hypothetical, dimensionless metric of “resolution failure,” where higher scores correspond to greater inflammatory persistence and a higher likelihood of developing post-viral NP.

Although not yet clinically validated, the proposed construct aligns with the translational goals of large-scale research initiatives, such as the NIH RECOVER consortium (facilitating access to PASC data through centralized platforms) [149] and the European IMI/IHI Inflammation/AI program [150], both aiming harmonize biomarker frameworks and identify molecular predictors of chronic post-viral symptoms.

Beyond biological modeling, AI-driven computational approaches offer complementary tools to capture the temporal dynamics of pain and inflammation. Recent machine-learning studies in pain medicine demonstrate that machine-learning models can predict pain trajectories and analgesic response [145,151,152]. For instance, a retrospective study in oncology in patients with colorectal cancer showed that integrating longitudinal pain data with biological variables improved the prediction of opioid responsiveness and interindividual pain trajectories [152].

Such approaches could be adapted to post-infectious or neuroinflammatory contexts to complement indices like the RFI, enabling dynamic, data-driven assessments of inflammatory persistence and nociceptive sensitization [35,146]. The RFI framework may ultimately facilitate patient stratification and guide the design of targeted interventions to restore inflammatory balance. By linking molecular pathways with clinical manifestations, it offers a translational tool for early detection and precision management of post-viral NP.

## 6. Conclusions

Persistent neuroinflammation following viral infection reflects a failure to achieve complete resolution of the immune response, whereby inadequate termination of inflammatory signaling perpetuates glial activation, oxidative stress, and neuronal hypersensitivity. This unresolved inflammatory state represents the critical biological interface linking post-infectious immune dysregulation to the development of NP.

Importantly, it is not the magnitude of the initial immune activation, but rather the insufficiency of SPMs and the loss of mitochondrial resilience that determine whether inflammation resolves or becomes chronic. Therapeutic strategies aimed at restoring this balance—through the enhancement of SPM biosynthesis, inhibition of NLRP3 inflammasome activity, and preservation of mitochondrial integrity—offer promising avenues for prevention and treatment.

The Resolution Failure Index (RFI) provides a conceptual and analytical framework for quantifying the imbalance between inflammation, oxidative stress, and resolution capacity, thereby capturing the biological continuum that sustains the persistence of NP. Although further validation in clinical settings is required, resolution-centered approaches hold potential for early identification of at-risk individuals and for guiding precision, mechanism-based neuroprotective interventions.

Ultimately, promoting the active resolution of inflammation—rather than merely suppressing it—emerges as a transformative paradigm for mitigating post-viral NP and restoring long-term neuroimmune homeostasis.

## Figures and Tables

**Table 1:** Experimental evidence linking defective efferocytosis to persistent macrophage and glial activation.

Author (Year)	Model	Mechanism Studied	Outcome (Persistent Activation)
Cai et al. (2019) [67]	Ischemic stroke (mouse)	Loss of STAT6/Arg1 signaling → impaired efferocytosis	Accumulation of apoptotic neurons, ↑ infarct size, neuroinflammation
Frank et al. (2018) [68]	Stress-induced (mouse)	↓ CD200R expression → microglial disinhibition	Microglial priming, exaggerated inflammatory responses, chronic glial activation
Kalinski et al. (2020) [52]	Sciatic nerve injury (mouse)	Macrophage efferocytosis of apoptotic leukocytes	Anti-inflammatory microenvironment, ↓ cytokine release, prevention of neuropathic pain
Kobayashi et al. (2020) [51]	Peripheral nerve injury (mouse)	↓ MerTK in M2 macrophages → defective efferocytosis	Sustained NF-κB activation, DAMP release, chronic nociceptive hypersensitivity
Soliman et al. (2023) [53]	Cortical injury (mouse)	EphA4 inhibits ERK/STAT6/MerTK signaling	Defective debris clearance, sustained microglial inflammasome activation
Song et al. (2021) [69]	SARS-CoV-2 infection (mouse)	Viral interference with apoptotic clearance	Persistent microglial activation, ↑ IL-6 and TNF-α
Wang et al. (2015) [37]	Alzheimer’s disease (APPPS1 mice)	TREM2 lipid sensing → efferocytosis of apoptotic neurons and amyloid debris	Defective efferocytosis → impaired microglial response, chronic neuroinflammation, accelerated neurodegeneration
Wanke et al. (2021) [54]	Murine and human macrophages	MerTK kinase activity inhibition	NLRP3 activation, unresolved inflammation
Zang et al. (2025) [70]	Thalamic hemorrhage (rat, CPSP model)	LXR-β activation → ↑ MerTK/Axl/CD36 via p-STAT6	Enhanced efferocytosis, ↓ neuroinflammation, alleviated post-stroke central pain

**Table 2:** Specialized pro-resolving mediators (SPMs): biosynthesis, mechanisms, neuroinflammation/neuropathic pain, and post-viral relevance.

SPM Family	Biosynthesis and Receptors	Core Mechanisms	Neuroinflammation/Neuropathic Pain	Post-Viral/COVID-19 Relevance
Lipoxins (LXA_4_, LXB_4_)	Arachidonic acid via 15-LOX/5-LOX; main receptor ALX/FPR2 [75].	Inhibits NLRP3, NF-κB, MAPK; lowers TNF-α/IL-1β/IL-6; supports epithelial repair [75].	LXA_4_ reduces glial activation and neuropathic pain; ALX/FPR2 signaling dampens microglial reactivity; nano-LXA_4_ improves cognition in neurodegeneration (summarized from preclinical studies).	ICU COVID-19 cohorts: low LXA_4_ despite severe disease; other SPMs rise but remain insufficient → impaired resolution [75,92].
Resolvins (RvD/E/T)	From EPA/DHA via 5/12/15-LOX; aspirin-acetylated COX-2 yields AT-resolvins; receptors ChemR23, ALX/FPR2, GPR32 [75].	Reduce neutrophil chemotaxis; increase phagocytosis/efferocytosis; lower IL-1β/IL-6/TNF-α; promote M2 polarization [75].	Limit microglial/astrocytic activation; constrain inflammasome; protect cognition in models (overview and synthesis).	Severe COVID-19: altered SPM profiles (↑RvE1, MaR2, RvD5; low LXA_4_), still inadequate resolution—candidate biomarkers/targets [75].
Protectins (PD1/NPD1/PDX)	DHA-derived (15-LOX); receptors include ALX/FPR2 [9].	Decrease oxidative stress and mitochondrial apoptosis; increase Iduna (DNA repair); stabilize BBB; promote neuro/angiogenesis [9].	PD1n-3 DPA lowers hippocampal IL-1β/TNF-α and reduces seizures; ALX/FPR2 and ChemR23 upregulated in epileptogenic astrocytes [109].	RSV: PCTR1/PD1 reduce viral load and lung inflammation, restore IFN-λ, induce cathelicidin [99]). COVID-19: higher plasma PD1 in critical illness associated with M2 polarization/IL-10 [100].
Maresins (MaR1/MaR2/MCTR)	DHA → 12-LOX in macrophages; receptors LGR6, ALX/FPR2 [74,104].	Enhance efferocytosis and tissue-repair programs; limit neutrophil influx/cytokines; MCTR couple clearance with regeneration [74,104].	MaR1 mitigates perioperative neuroinflammation/cognitive decline [110]; promotes axonal regrowth; dampens spinal glia and TRPV1/PI3K-AKT-mTOR [107]; provides long-lasting analgesia via NF-κB/CGRP control [71]. MaR2 (intrathecal) reduces orofacial nociception, prevents postoperative hyperalgesia, and reverses trigeminal NP by suppressing c-Fos and NF-κB^+^/CGRP^+^ TG neurons [108].	Post-COVID syndrome: 12-week SPM-enriched marine oil increased 14-HDHA/17-HDHA/18-HEPE and improved fatigue/dyspnea—supporting translational potential [105].

Abbreviations: LOX, lipoxygenase; COX-2, cyclooxygenase-2; BBB, blood–brain barrier;TG, trigeminal ganglion.

## Data Availability

No new data were created or analyzed in this study. Data sharing is not applicable to this article.

## Abbreviations

The following abbreviations are used in this manuscript:

ADAM17	A disintegrin and metalloproteinase 17
AMPK	AMP-activated protein kinase
APPPS1	Amyloid Precursor Protein/Presenilin-1 mouse model
ATP	Adenosine triphosphate
Axl	Axl receptor tyrosine kinase
BBB	Blood–brain barrier
BDNF	Brain-derived neurotrophic factor
BCL-2	B-cell lymphoma 2
CGRP	Calcitonin gene-related peptide
cf-mtDNA	Cell-free mitochondrial DNA
COX-2	Cyclooxygenase-2
CRP	C-reactive protein
DAMPs	Damage-associated molecular patterns
DAP12	DNAX-activating protein of 12 kDa
DHA	Docosahexaenoic acid
DRG	Dorsal root ganglion
EPA	Eicosapentaenoic acid
ERK	Extracellular signal-regulated kinase
GFAP	Glial fibrillary acidic protein
GPR32	G protein-coupled receptor 32
HMGB1	High mobility group box 1
hs-CRP	High-sensitivity C-reactive protein
Iduna	Induced DNA damage response protein 1
IFN-λ	Interferon lambda
IL	Interleukin
iNOS	Inducible nitric oxide synthase
LGR6	Leucine-rich repeat-containing G protein-coupled receptor 6
LOX	Lipoxygenase
LPA	Lysophosphatidic acid
LPS	Lipopolysaccharide
LXA_4_, LXB_4_	Lipoxin A_4_, Lipoxin B_4_
MAPK	Mitogen-activated protein kinase
MaR1, MaR2	Maresin 1, Maresin 2
MCTR	Maresin conjugates in tissue regeneration
MDA	Malondialdehyde
MerTK	Mer receptor tyrosine kinase
MFG-E8	Milk fat globule epidermal growth factor 8
mtROS	Mitochondrial reactive oxygen species
NAC	N-acetylcysteine
NET	Neutrophil extracellular trap
Neuro-PASC	Neurologic Post-Acute Sequelae of SARS-CoV-2 infection
NF-κB	Nuclear factor kappa-light-chain-enhancer of activated B cells
NLRP3	NOD-like receptor family pyrin domain-containing 3
NP	Neuropathic pain
NPD1	Neuroprotectin D1
Nrf2	Nuclear factor erythroid 2–related factor 2
PCTR1	Protectin conjugate in tissue regeneration 1
PD1, PDX	Protectin D1, Protectin DX
PI3K	Phosphoinositide 3-kinase
PPARγ	Peroxisome proliferator-activated receptor gamma
PS	Phosphatidylserine
Rac1	Ras-related C3 botulinum toxin substrate 1
RA	Rheumatoid arthritis
RAGE	Receptor for advanced glycation end-products
RFI	Resolution Failure Index
ROS	Reactive oxygen species
RSV	Respiratory Syncytial Virus
RvD1, RvD5, RvE1	Resolvin D1, Resolvin D5, Resolvin E1
RXRα	Retinoid X receptor alpha
SOCS3	Suppressor of cytokine signaling 3
MS	Multiple Sclerosis
SPM, SPMs	Specialized pro-resolving mediator(s)
STAT6	Signal transducer and activator of transcription 6
TGF-β	Transforming growth factor beta
TG	Trigeminal ganglion
TREM2	Triggering receptor expressed on myeloid cells 2
TRPV1	Transient receptor potential vanilloid 1
UCP2	Uncoupling protein 2
